# Rapid and Simple Morphological Assay for Determination of Susceptibility/Resistance to Combined Ciprofloxacin and Ampicillin, Independently, in *Escherichia coli*

**DOI:** 10.3390/antibiotics13070676

**Published:** 2024-07-20

**Authors:** Isidoro López, Fátima Otero, María del Carmen Fernández, Germán Bou, Jaime Gosálvez, José Luis Fernández

**Affiliations:** 1 Genetics Unit, Institute of Biomedical Research of A Coruña (INIBIC)—Complejo Hospitalario Universitario A Coruña (CHUAC), 15006 A Coruña, Spain; isidoro.lopez.baltar@sergas.es (I.L.); fatima.otero.farina@sergas.es (F.O.); 2 Molecular Genetics and Radiobiology Laboratory, Centro Oncológico de Galicia, 15009 A Coruña, Spain; 3 CIBER (Biomedical Research Networking Centre) de Enfermedades Infecciosas (CIBERINFEC), Instituto de Salud Carlos III, 28029 Madrid, Spain; ma.carmen.fernandez.lopez@sergas.es (M.d.C.F.); german.bou.arevalo@sergas.es (G.B.); 4 Microbiology Service and INIBIC—Complejo Hospitalario Universitario A Coruña (CHUAC), 15006 A Coruña, Spain; 5 Genetics Unit, Facultad de Biología, Universidad Autónoma de Madrid, 28049 Madrid, Spain; jaime.gosalvez@gmail.com

**Keywords:** antibiotic combination, bacterial nucleoid, DNA fragments, rapid antibiogram, quinolone, beta-lactam

## Abstract

Current antibiograms cannot discern the particular effect of a specific antibiotic when the bacteria are incubated with a mixture of antibiotics. To prove that this task is achievable, *Escherichia coli* strains were treated with ciprofloxacin for 45 min, immobilized on a slide and stained with SYBR Gold. In susceptible strains, the nucleoid relative surface started to decrease near the MIC, being progressively condensed as the dose increased. The shrinkage level correlated with the DNA fragmentation degree. Ciprofloxacin-resistant bacilli showed no change. Additionally, *E. coli* strains were incubated with ampicillin for 45 min and processed similarly. The ampicillin-susceptible strain revealed intercellular DNA fragments that increased with dose, unlike the resistant strain. Co-incubation with both antibiotics revealed that ampicillin did not modify the nucleoid condensation effect of ciprofloxacin, whereas the quinolone partially decreased the background of DNA fragments induced by ampicillin. Sixty clinical isolates, with different combinations of susceptibility-resistance to each antibiotic, were co-incubated with the EUCAST breakpoints of susceptibility of ciprofloxacin and ampicillin. The morphological assay correctly categorized all the strains for each antibiotic in 60 min, demonstrating the feasible independent evaluation of a mixture of quinolone and beta-lactam. The rapid phenotypic assay may shorten the incubation times and necessary microbial mass currently required for evaluation.

## 1. Introduction

Hospital-acquired infections, usually in the intensive care unit (ICU), cause great concern worldwide since they result in a high mortality and morbidity rate, mainly in immunocompromised patients. These critical patients require rapid and effective antibiotic treatment [1]. The development of a rapid antibiogram is a great challenge that can save lives and reduce health care costs [2,3,4].

Microbiology laboratories perform antibiograms following standardized procedures, based on the determination of the level of inhibition exerted by a given antibiotic on bacterial growth [5]. The assays require incubation based on the time necessary for the microorganisms to grow, involving periods from 24 h to several days [5,6]. 

New methodologies are being developed for rapid antibiotic-susceptibility testing as reviewed in [6,7,8,9,10]. Most current techniques are based on the evaluation of microbial proliferation, for example, using automated methods that accelerate the achievement of results. Thus, panels have been introduced in the market measuring turbidity changes or in which the culture medium includes a fluorescent indicator, which allows for results to be obtained in about eight hours. 

Microbial growth results can also be accelerated using real-time microscopy [10,11], light scattering with a laser beam [12] and real-time quantitative polymerase chain reaction (qPCR) of specific bacterial DNA sequences [13]. In this way, it is possible to obtain results of the potential effect on bacterial growth after 6 h of culture with the antibiotic [13]. Another procedure relies on the evaluation of cell mass and growth by vibrating devices [14], the changes in a rotating magnetic field [15], loss of nanometer oscillations [16] or use of recombinant bacteriophages, which produce light when the luciferase gene expression is triggered once they infect a specific bacterial host [17].

In addition to the evaluation of bacterial growth, some experimental approaches are being developed based on the effect, or lack thereof, of certain physiological or metabolic parameters by antibiotics like NADH and FADH, pH, heat, membrane potentials or cell electrophysiology that behave as biochemical flags of active cellular metabolism [18,19,20,21]. Proteomic and other “omic” methodologies and the use of next-generation sequencing are also under development, although several issues remain to be resolved [9,22]. 

We had previously developed a fast and simple methodology to determine the resistance to quinolones [23,24]. The bacteria incubated with the antibiotic are included in an inert microgel on a slide and incubated with a lysis solution that removes the cell wall, releasing the nucleoids. This DNA is stained with a high-sensitivity fluorochrome and is observed under fluorescence microscopy. Since the quinolones induce the fragmentation of bacterial DNA, if the strain is susceptible, a halo of DNA fragments that have diffused from the residual bacterial body is observed. If the strain is resistant, the nucleoids appear compact. 

Subsequently, this technology was adapted for antibiotics that inhibit the synthesis of peptidoglycan, such as beta-lactams and glycopeptides [25,26]. In this case, the lysis solution only acts on those bacteria whose cell wall has been affected by the antibiotic, releasing the nucleoid. Resistant strains are not affected at the cell wall by the lysis solution and maintain their usual appearance. These methodologies have also allowed the rapid determination of colistin resistance [27]. The most significant achievement of this methodology is that the results can be obtained in two hours. 

The antibiograms based on cell growth or in modification of physiological or metabolic targets cannot discriminate between the susceptibility/resistance of each specific antibiotic when incubated simultaneously in combination. This means that the sensitivity or resistance to a quinolone and a beta-lactam can only be determined separately. If the combination affects bacterial growth, it is not possible to discern whether this has been the effect of both antibiotics or only one of them, nor which one. It also happens with the microscopic techniques that we have developed for quinolones and beta-lactams, since the different lysing solutions used are not compatible. With this aim, a different methodology is needed with the goal to assess synergism, indifference or antagonism between two antibiotics against a specific species of bacteria. Regardless of the technique used, an additional 18–24 h of incubation is required to obtain a result.

Here, we report the feasibility of a rapid procedure to determine the susceptibility/resistance to the combination of ciprofloxacin and ampicillin in *E. coli* strains, based on a simple microscope visualization. This aforementioned procedure allows one to simultaneously discriminate the effect, or lack thereof, of each antibiotic separately.

## 2. Results

### 2.1. Dose-Response to Ciprofloxacin 

**Figure 1:**
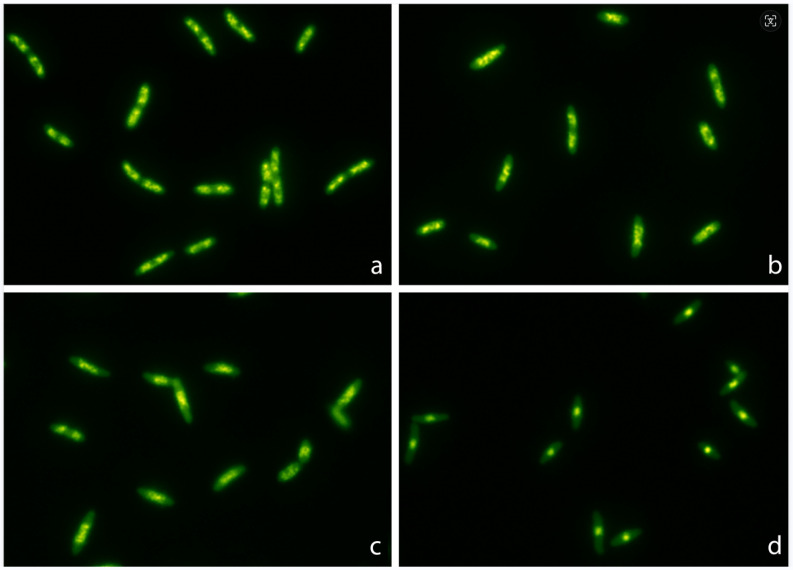
Representative images of *Escherichia coli* TG1 strain incubated with increasing concentrations of ciprofloxacin (MIC: 0.012 mg/L) for 45 min and stained with SYBR Gold. The nucleoids were progressively condensed at the middle of the bacteria as the concentration of ciprofloxacin increased. The median relative nucleoid surface (RNS) with respect to the total surface of the bacteria was 46.9% (42.03–50.33) for 0 mg/L (**a**), 41.2% (37.85–44.38) for 0.012 mg/L (**b**), 28.7% (26.23–30.95) for 0.04 mg/L (**c**) and 13.4% (11.95–14.85) for 0.5 mg/L (**d**).

**Figure 2:**
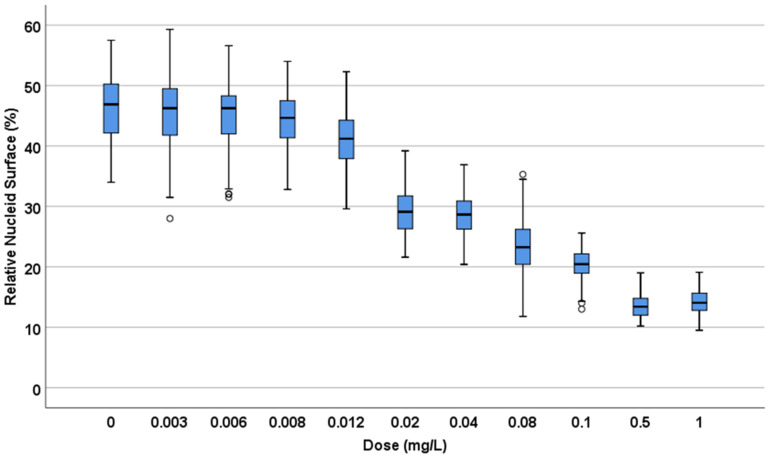
Relative nucleoid surface (RNS) of *E. coli* TG1 strain incubated with increasing concentrations of ciprofloxacin (MIC: 0.012 mg/L) for 45 min. The data are presented as box and whisker plots. The horizontal line in the box indicates the median, the lower line of the box is the first quartile, the upper line of the box is the third quartile and the whiskers (the end of the vertical lines) are the maximum and minimum values. Abnormal values are presented as dots outside the box.

**A.** *E. coli* TG1 strain was incubated with increasing concentrations of ciprofloxacin for 45 min and processed to visualize the nucleoids inside the bacteria (Figure 1). In the control untreated culture, the baseline relative nucleoid surface (RNS) occupied 46.9% (42.03–50.33) (median and inter quartile range IQR: Q1–Q3) of the total surface of the bacteria. The RNS was significantly lower at the MIC, and the nucleoids were progressively condensed at the middle of the bacteria as the concentration of ciprofloxacin increased, so that after 0.5–1 mg/L, the median (IQR) RNS was stabilized at 13.4 (11.95–14.85)–14% (12.80–15.68), respectively (Figure 2).

**Figure 3:**
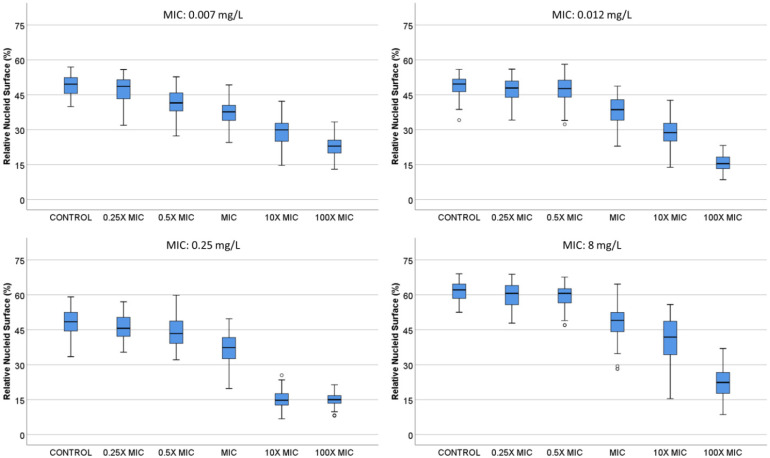
Relative nucleoid surface (RNS) of *E. coli* strains with MIC 0.007, 0.012, 0.25 and 8 mg/L, incubated with ciprofloxacin at concentrations that are multiples of MIC.

**B.** *E. coli* strains with MIC 0.007, 0.012, 0.25 and 8 mg/L were incubated with 0, 0.25×, 0.5×, 1×, 10× and 100× MIC concentrations of ciprofloxacin (Figure 3). In general, the nucleoids occupied around 50% of the total surface of the bacteria in the control untreated samples. The nucleoids started to condense at 1× MIC, being progressively more compact at 10× and 100× MIC doses, finally achieving a 15.0 (13.43–16.78)–22.9% (19.93–25.55) median (IQR) RNS at the center of the bacteria. In the strain with lower MIC, the effect was already significant with the 0.5× MIC dose. 

In a previous report, these strains had also been incubated with a lysing solution to remove the cell wall and release the nucleoids in the microgel [23]. Ciprofloxacin induced chromosomal DNA breakage resulting in a peripheral halo of DNA fragments that diffused from the central residual bacteria. The higher the dose, the greater the fragmentation, the greater the number of DNA spots and the greater the circular surface area of diffusion. The RNS was strongly correlated with the diameter of dispersion of DNA spots observed after bacterial lysis (Spearman’s rho: −0.83, *p* < 0.001) (Supplementary Figure S1). The higher the fragmentation, the lower the RNS, i.e., the higher the nucleoid compaction.

### 2.2. Dose-Response to Ampicillin

**Figure 4:**
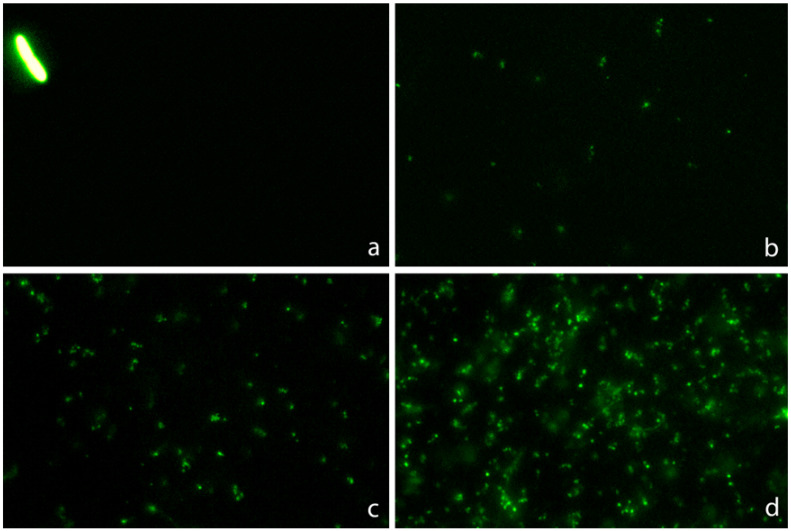
Images of intercellular DNA fragments after incubation for 45 min with increasing concentrations of ampicillin in a susceptible *E. coli* strain (MIC: 4 mg/L). The median DNA fragments per µm^2^ were 0.03 (0.007–0.056) for 0 mg/L (**a**), 0.57 (0.42–0.75) for 2 mg/L (**b**), 1.40 (0.89–1.59) for 4 mg/L (**c**) and 2.28 (2.06–2.54) for 16 mg/L (**d**).

**Figure 5:**
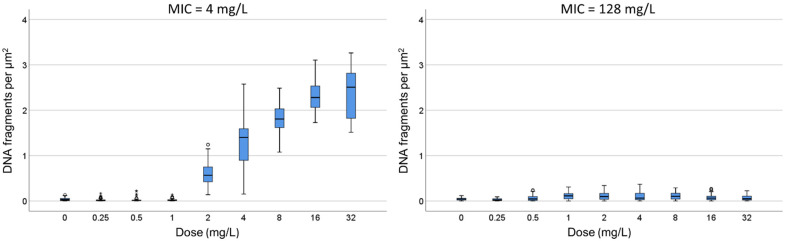
Intercellular DNA fragments per µm^2^ after incubation for 45 min with increasing concentrations of ampicillin in one susceptible *E. coli* strain (MIC: 4 mg/L) and one resistant (MIC: 128 mg/L). The horizontal line in the box indicates the median, the lower line of the box is the first quartile, the upper line of the box is the third quartile and the whiskers (the end of the vertical lines) are the maximum and minimum values. Abnormal and extreme abnormal values are presented as dots and asterisk, respectively, outside the box.

**C.** One strain susceptible (MIC: 4 mg/L) and one resistant (MIC: 128 mg/L) were incubated with increasing concentrations of ampicillin for 45 min (Figure 4). In the susceptible strain, a significant increase in the intercellular DNA spots was evident at 2 mg/L, rising progressively to achieve a median (IQR) of 2.51 spots/µm^2^ (1.82–2.82) at 32 mg/L. The resistant strain did not increase the background DNA fragments at any dose (Figure 5).

### 2.3. Dose-Responses to Combined Ciprofloxacin and Ampicillin

One strain susceptible to both ciprofloxacin (MIC: 0.047 mg/L) and ampicillin (MIC: 4 mg/L) and another strain resistant to ciprofloxacin (MIC: 8 mg/L) but susceptible to ampicillin (MIC: 6 mg/L) were co-incubated with both antibiotics for 45 min. After technical processing, the RNS and background of DNA fragments were recorded.

**Figure 6:**
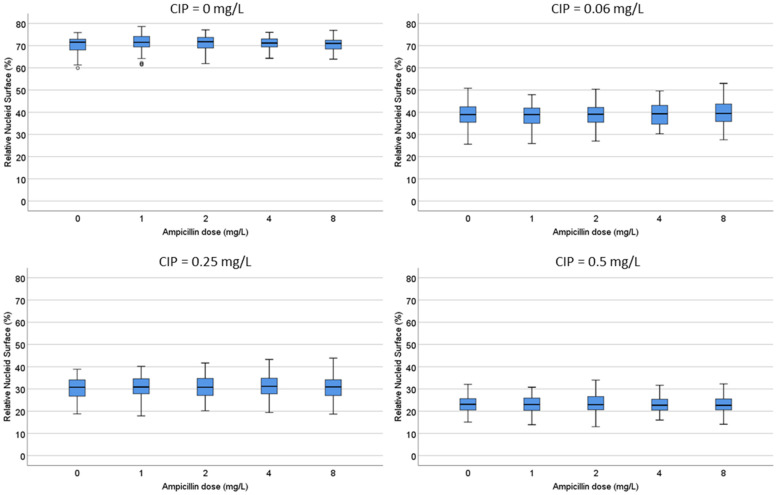
Evaluation of the influence of ampicillin on the relative nucleoid surface (RNS) effect of ciprofloxacin. One *E. coli* strain susceptible to ciprofloxacin (MIC: 0.047 mg/L) and ampicillin (MIC: 4 mg/L) was incubated with fixed concentrations of ciprofloxacin (0, 0.06, 0.25 or 0.5 mg/L) together with increasing concentrations of ampicillin (0, 1, 2, 4 and 8 mg/L). Numerical values are presented in Supplementary Table S1.

**D.** Both strains were incubated with ciprofloxacin at 0, 0.06, 0.25 and 0.5 mg/L. For each fixed dose of ciprofloxacin, ampicillin was simultaneously added at 0, 1, 2, 4 and 8 mg/L. In the strain susceptible to ciprofloxacin, the median (IQR) RNS decreased from 71.55% (68.03–72.90) to 20.5% (18.23–22.70) as the quinolone increased. The RNS from each ciprofloxacin dose was not modified by any concentration of ampicillin (Figure 6; Supplementary Table S1). In the strain resistant to ciprofloxacin, the baseline RNS was not affected by the quinolone nor by the ampicillin. 

**Figure 7:**
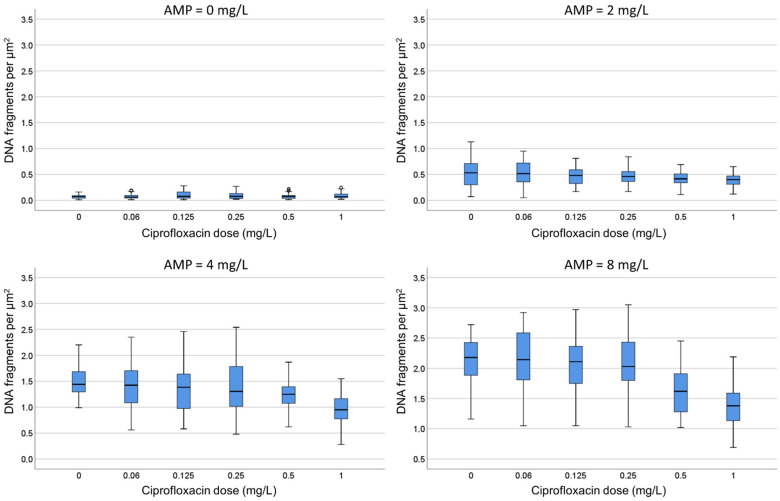
Evaluation of the influence of ciprofloxacin on the intercellular DNA fragments per µm^2^ produced by ampicillin (AMP). The *E. coli* strain susceptible to ciprofloxacin (MIC: 0.047 mg/L) and ampicillin (MIC: 4 mg/L) was incubated with fixed concentrations of ampicillin (0, 2, 4 or 8 mg/L) together with increasing concentrations of ciprofloxacin (0, 0.06, 0.125, 0.25, 0.5 and 1 mg/L). Numerical values are presented in Supplementary Table S2.

**E.** Both strains were incubated with ampicillin at 0, 2, 4 and 8 mg/L. For each fixed dose of ampicillin, ciprofloxacin was simultaneously added at 0, 0.06, 0.125, 0.25, 0.5 and 1 mg/L. In the strain susceptible to ciprofloxacin, as ampicillin increased, the median (IQR) of background DNA fragments increased from 0.07 (0.04–0.09) to 2.18 (1.88–2.43) spots/µm^2^. For each concentration of ampicillin, ciprofloxacin decreased the spots/µm^2^ in a dose-dependent way (Figure 7; Supplementary Table S2). For example, the 2.18 (1.88–2.43) spots/µm^2^ obtained at the highest dose of ampicillin alone were reduced to 1.38 (1.13–1.60) spots/µm^2^ when co-incubated with the highest dose of the quinolone. Despite the reduction in the DNA spots with respect to the culture incubated with ampicillin only, the background continued to be highly remarkable. In the strain resistant to ciprofloxacin, the co-incubation with the quinolone did not affect the background of DNA spots produced by ampicillin.

### 2.4. Comparison with the Standard Antibiogram

**F.** *E. coli* clinical isolates (n = 60) were categorized accordingly to the standard antibiograms as susceptible to ciprofloxacin and ampicillin (n = 13), non-susceptible to both antibiotics (n = 18), susceptible to ciprofloxacin and non-susceptible to ampicillin (n = 11) and non-susceptible to ciprofloxacin and susceptible to ampicillin (n = 18). These strains were co-incubated with the EUCAST breakpoints of susceptibility for ciprofloxacin (0.25 mg/L) and ampicillin (8 mg/L) for 45 min and technically processed [28]. 

An RNS breakpoint for ciprofloxacin was established based on one strain whose MIC was similar to that of the CLSI cut-off point. This strain had a baseline median (IQR) RNS value of 48.4% (44.50–50.27), which decreased significantly to 37.3% (32.52–41.61) after being incubated with the MIC-CLSI breakpoint dose. The TG1 baseline median (IQR) RNS was the lowest, 46.9% (42.03–50.33). Taking this into account, a cut-off point of susceptibility for 0.25 mg/L ciprofloxacin was established as ≤40% median RNS. 

Regarding ampicillin, in strains whose MIC was close to the CLSI breakpoint, this dose produced a median (IQR) of 2.1 (1.95–2.43) spots/µm^2^ of intercellular DNA fragments, which decreased to 1.7 (1.24–2.32) spots/µm^2^ when co-incubated with the CLSI breakpoint for ciprofloxacin, being highly susceptible to the quinolone. The background of the strains was habitually very low, 0.01 (0.007–0.030)–0.05 (0.007–0.138) spots/µm^2^ median (IQR), but one strain showed 0.5 (0.30–0.71) spots/µm^2^, so a cut-off point of susceptibility for 8 mg/L ampicillin was established as ≥1 spots/µm^2^ median.

**Figure 8:**
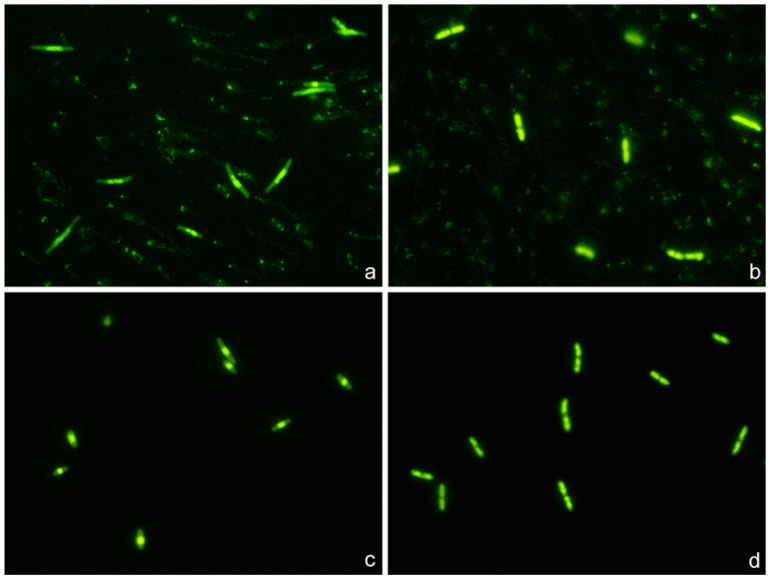
Representative images of the technique for the rapid detection of susceptibility or not to a quinolone and a beta-lactam simultaneously. Four *E. coli* strains were incubated with the EUCAST breakpoint concentrations of susceptibility for ciprofloxacin (0.25 mg/L) and ampicillin (8 mg/L), simultaneously for 45 min. The fluorescence microscopy images were obtained after technical processing. (**a**) Strain susceptible to both antibiotics (MIC ciprofloxacin: 0.012 mg/L; MIC ampicillin: 2 mg/L). The visualization of an intense central spot corresponding to a condensed nucleoid inside the bacteria is the marker of susceptibility to ciprofloxacin. The presence of a background of DNA fragments is indicative of susceptibility to ampicillin. (**b**) Strain non-susceptible to ciprofloxacin and susceptible to ampicillin (MIC ciprofloxacin: 8 mg/L; MIC ampicillin: 4 mg/L). The fluorescence from the nucleoid is spread inside the bacteria, and no central spot is evident. This is the pattern observed in the control *E. coli*, without quinolone treatment, thus evidencing resistance to the ciprofloxacin dose. Otherwise, the presence of background DNA fragments is the signal of susceptibility to ampicillin. (**c**) Strain susceptible to ciprofloxacin and non-susceptible to ampicillin (MIC ciprofloxacin: 0.047 mg/L; MIC ampicillin: 12 mg/L). The fluorescence inside the bacteria is concentrated in a central spot, being indicative of affectation by the quinolone. There is no background of DNA fragments, so the strain is resistant to the ampicillin dose. (**d**) Strain non-susceptible to both antibiotics (MIC ciprofloxacin: 4 mg/L; MIC ampicillin: 12 mg/L). The fluorescence from the nucleoid is spread inside the bacteria, so the strain is resistant to the ciprofloxacin dose. No background of DNA fragments is observed, so the strain is also resistant to the ampicillin dose.

Strains susceptible to ciprofloxacin and ampicillin clearly showed highly condensed central nucleoids and a significant background of intercellular DNA spots (Figure 8). Strains susceptible to ciprofloxacin but non-susceptible to ampicillin revealed highly packed nucleoids but practically no background of DNA spots. Strains non-susceptible to ciprofloxacin but susceptible to ampicillin showed a very high background of DNA fragments, but the nucleoids were spread inside the cells, without change with respect to those of the control untreated cultures. Strains non-susceptible to both antibiotics were similar to the control untreated cultures, with spread nucleoids and practically no intercellular DNA fragments (Figure 8). The results of the categorization by the phenotypic functional assay were fully concordant with those from the standard antibiogram for each specific antibiotic.

## 3. Discussion

Progress has been achieved in the isolation and rapid identification of the microorganism that causes an infection, through specific selective cultures and molecular or proteomic techniques. Nevertheless, after identification, an antibiogram must be performed. Many experimental rapid antibiograms rely on the effect, or lack thereof, by the antibiotics on the bacterial growth or related metabolic activity and cell mass parameters [5,6,7,8,9,10]. To perform these antibiograms it is necessary to collect a sufficient mass of bacteria (i.e., germs) to evaluate. The more antibiotics to test, the more germs, and all of this implies a necessary and preceding growth time. 

The main mechanisms of antibiotic resistance include neutralization through enzymatic activity, modification of antibiotic targets, inactivation of porin channels and upregulation of efflux pumps [29]. Nucleic acid or mass-spectroscopy-based approaches may detect mutations in known genes or specific metabolites, or the proteins involved in the different mechanisms of resistance, so theoretically may provide information on possible susceptibility/resistance to different antibiotics in a single assay [9]. Nevertheless, they require previous knowledge of the resistance mechanism and may not be confident in evaluating the level of non-susceptibility and detecting uncharacterized processes of resistance. Moreover, the molecular profile may not be strictly correlated with the functional phenotype [7]. Phenotypic assays overcome these limitations. 

Regarding morphological phenotypic responses, it has been reported that the treatment of *E. coli* with fluoroquinolones like fleroxacin produces nucleoids that are progressively smaller with increasing concentrations [30]. This is extensively explored in the present report, detailing quantitatively that *E. coli* exposed to ciprofloxacin shows a marked reorganization of the nucleoid when it is affected by the antibiotic. However, resistant bacilli show no change in the morphological pattern of the nucleoid, being similar to the control without quinolone. The phenomenon follows a dose-response behavior, depending on the MIC of the pathogen. This quinolone evaluation system is a much simpler and faster technique than the one we had initially developed, which needed a bacterial lysis and the evaluation of the level of fragmentation or not from the DNA of the released nucleoids [23,24]. In the new technique, it is not necessary to perform any lysis. Otherwise, regarding phenotypic responses to beta-lactams, we had stated that Gram-negative bacilli incubated with penicillin resulted in a background of intercellular DNA fragments which seemed related to dose and susceptibility [25]. 

Taking advantage of these referred morphological responses, the present report is a proof of concept of the feasibility of an entirely phenotypic functional assay for the rapid simultaneous determination of susceptibility or not to a mixture of a quinolone (ciprofloxacin) and a penicillin (ampicillin) in *E. coli*, independent of the possible mechanism of resistance. The experimental conditions were set up, introducing quantitative estimations through image analysis, validating the combination of both parameters to assess each antibiotic individually. This is a simple methodology, summarized in the following steps: 1. Brief co-incubation of the bacterial strain with both antibiotics in broth culture; 2. Immobilization of the microorganisms and background DNA fragments of the culture on a slide; 3. High-sensitivity fluorescent nucleic acid dying for microscopy and evaluation of the condensation or not of the nucleoid inside the bacteria (regarding ciprofloxacin susceptibility or resistance, respectively) and presence or not of intercellular DNA spots (regarding ampicillin susceptibility or resistance, respectively). It must be noted that ciprofloxacin and/or ampicillin treatments did not significantly modify the cell length or volume under the incubation time and conditions of the assay.

In the present study, the technical manipulation is minimum, with practically only incubation and visualization, so results may be obtained in one hour. It must be taken into account that if the bacteria are not growing exponentially, they should be previously incubated in liquid broth for 90 min to achieve the active growing phase. A simple brief visual inspection of the slide allows one to confidently recognize the activity, or lack thereof, of each antibiotic. If a more precise assessment is required, the use of image analysis software may provide an accurate quantitative approach, as demonstrated. To gain assurance, it is recommended the concurrent processing of known reference strains, susceptible to both antibiotics, resistant to both, susceptible to one and resistant to another and vice versa. 

Preliminary results evidenced the nucleoid condensation effect by ciprofloxacin and the presence of a background of DNA fragments induced by beta-lactams like penicillin, amoxicillin and imipenem in several species of Gram-negative bacilli. Further experiments will expand the feasibility of the combined phenotypic assay.

Although the work is a proof of concept, it may have clinical interest since some studies have shown that the combination of a fluoroquinolone with a beta-lactam had in vitro and in vivo synergy against extended-spectrum beta-lactamase producing *E. coli* and *P. aeruginosa* isolates [31]. Moreover, it has reduced mortality for bacteremia caused by Gram-negative bacilli [32].

The possibility of being able to incubate bacteria with a combination of antibiotics and recognize the sensitivity or resistance to each of them, simultaneously but independently, can reduce the required cell amounts for testing and shorten the time to obtain results. Such aforementioned procedural improvements may be crucial for the success of the treatment in cases of great urgency. In addition, it could also guide the very urgent prescription of a combination of antibiotics to a critical patient in order to obtain antibacterial synergism or try to reduce the toxicity of a certain drug. This may also help to prevent the development and spread of antimicrobial resistance. 

## 4. Materials and Methods

### 4.1. Bacterial Strains and Minimum Inhibitory Concentration (MIC) Determination 

The work was performed using *E. coli* TG1 strain and clinical isolates of *E. coli* (n = 60) collected at the University Hospital A Coruña between 2010 and 2021. Bacteria were routinely grown in Mueller-Hinton broth (1% Bacto Tryptone, 0.5% yeast extract, 0.5% NaCl) (Merck KGaA, Darmstadt, Germany) or on Mueller-Hinton agar plates (BD, New Jersey, US), at 37 °C in aerobic conditions. Cell growth in liquid cultures was observed by monitoring turbidity with a spectrophotometer at OD600 (Unicam 8625, Cambridge, UK). The MICs were ascertained by automated microdilution (MicroScan Walkaway, Siemens, Munich, Germany) and corroborated by a MIC Test Strip (Liofilmchem, Roseto degli Abruzzi (TE), Italy) according to the manufacturer’s instructions, with susceptibility defined using EUCAST criteria [28]. 

### 4.2. Technical Processing 

To immobilize the intact bacteria on slides, the Micro-Halomax^®^ kit for fluorescence microscopy (Halotech DNA SL, Madrid, Spain) was used [23,24,25]. An aliquot of each sample was diluted to a concentration of 1 × 10^7^ microorganisms/mL in Mueller-Hinton broth. A total of 18 μL of bacterial culture was mixed with 6 μL of the antibiotic solution in a microtiter plate, the final volume being adjusted to 30 μL and incubated 45 min at 37 °C. In addition, 0.5 mL snap cap microfuge tubes containing gelled aliquots of low-melting-point agarose are provided with the kit to process a sample. The tube was placed in a water bath at 90–100 °C for 5 min to completely melt the agarose, and then placed in a water bath at 37 °C. The 30 μL of the bacterial–antibiotic sample was added to the tube and mixed with the 70 μL of melted agarose. Aliquots of 10 μL of the sample–agarose mixture were pipetted onto a precoated slide and covered with an 18 × 18 mm coverslip. The slide was placed on a cold plate in a refrigerator (4 °C) for 5 min, to allow the agarose to produce a microgel with the trapped intact cells inside. The slide was dehydrated by incubating horizontally in increasing cold (−20 °C) ethanol baths (70–90–100%) for 2 min each and air-dried in an oven. DNA staining was performed with 25 μL of the fluorochrome SYBR Gold (Molecular Probes, Eugene, OR, USA) that had been diluted at 1:400 in TBE buffer (0.09 M Tris-Borate, 0.002 M EDTA, pH 7.5), incubating for 5 min in the dark. 

Images were viewed under an epifluorescence microscope (Nikon E800, Tokyo, Japan), with a 100× objective and appropriate fluorescence filter, and acquired using a high-sensitivity CCD camera (KX32ME, Apogee Instruments, Roseville, CA, USA). Groups of 16-bit digital images were obtained for each experimental point under similar conditions and stored in a tiff. file format. Image analysis was performed using macros designed with NIS-Elements software (https://www.microscope.healthcare.nikon.com/products/software/nis-elements, Nikon Instruments Inc., Tokyo, Japan). For ciprofloxacin, the total cell surface and nucleoid surface to establish the relative nucleoid surface (RNS, in %) were measured in 100 cells per assay. For ampicillin, the software discriminates the bacteria from spots corresponding to DNA fragments spread out in the background and counts the number of these particles. Results were reported as DNA fragments per µm^2^ of slide surface. 

### 4.3. Experiments 

#### 4.3.1. Dose-Response to Ciprofloxacin 

A. *E. coli* TG1 strain exponentially growing in Mueller-Hinton broth was incubated with ciprofloxacin (MIC: 0.012 mg/L) for 45 min, to eleven increasing concentrations from 0.003 to 1 mg/L, besides the control without antibiotic. After technical processing, the RNS was established for each concentration.

B. Strains with MIC 0.007, 0.012, 0.25 and 8 mg/L were incubated with 0, 0.25×, 0.5×, 1×, 10× and 100× MIC concentrations, for each strain. After technical processing, the RNS was established for each concentration. Previous data about the degree of DNA fragmentation were available from experiment B [23], where the diameter of the halo of DNA spots emerging from the residual core of the lysed bacteria had been measured in μm. This allowed to estimate a possible correlation between RNS and DNA fragmentation level, as well as regression analysis. 

#### 4.3.2. Dose-Response to Ampicillin

C. One strain susceptible (MIC: 4 mg/L) and one resistant (MIC: 128 mg/L) were incubated for 45 min with 0, 0.25, 0.5, 1, 2, 4, 8, 16 and 32 mg/L ampicillin. After technical processing, the number of intercellular DNA spots per µm^2^ was established.

#### 4.3.3. Dose-Responses to Combined Ciprofloxacin and Ampicillin

This experiment was performed with two strains, one susceptible to both ciprofloxacin (MIC: 0.047 mg/L) and ampicillin (MIC: 4 mg/L) and another strain resistant to ciprofloxacin (MIC: 8 mg/L) but susceptible to ampicillin (MIC: 6 mg/L). After 45 min of co-incubation and subsequent technical processing, the RNS and background of DNA fragments were recorded.

D. Both strains were incubated with ciprofloxacin at 0, 0.06, 0.25 and 0.5 mg/L. For each fixed dose of ciprofloxacin, ampicillin was simultaneously added at 0, 1, 2, 4 and 8 mg/L. 

E. Both strains were incubated with ampicillin at 0, 2, 4 and 8 mg/L. For each fixed dose of ampicillin, ciprofloxacin was simultaneously added at 0, 0.06, 0.125, 0.25, 0.5 and 1 mg/L. 

#### 4.3.4. Comparison with the Standard Antibiogram

F. Clinically isolated *E. coli* strains (n = 60) were co-incubated with the EUCAST breakpoints of susceptibility for ciprofloxacin (0.25 mg/L) and ampicillin (8 mg/L) for 45 min. After technical processing, susceptibility or non-susceptibility was determined according to the RNS for ciprofloxacin and the number of intercellular DNA spots per µm^2^ for ampicillin.

### 4.4. Data Analysis

Data were analyzed with the SPSS 26 software package for Windows (IBM). A Friedman test and Wilcoxon text with Bonferroni correction were employed for statistical comparisons. The relationship between RNS and DNA fragmentation was assessed using Spearman’s correlation and linear regression analysis. Significance was established as *p* < 0.05. 

## Data Availability

Not all data are contained in the manuscript and supplementary material. Data are contained within the article and Supplementary Materials.

## Supplementary Materials

The following supporting information can be downloaded at https://www.mdpi.com/article/10.3390/antibiotics13070676/s1, Figure S1: Linear regression showing the relationship between the mean relative nucleoid surface (RNS) of *E. coli* strains incubated with ciprofloxacin and the mean diameter of dispersion of DNA spots observed after bacterial lysis [23], Table S1: Numerical values corresponding to Figure 6, Table S2: Numerical values corresponding to Figure 7.
