# Supplementary material for: Rapid and Simple Morphological Assay for Determination of Susceptibility/Resistance to Combined Ciprofloxacin and Ampicillin, Independently, in Escherichia coli
Source: Antibiotics (Basel). 2024 Jul 20;13(7):676. doi: 10.3390/antibiotics13070676 (PMC11273673; doi:10.3390/antibiotics13070676)
Supplement: Supplementary file 1 [file antibiotics-13-00676-s001.zip › antibiotics-3053478-supplementary.pdf]

Supplementary Materials

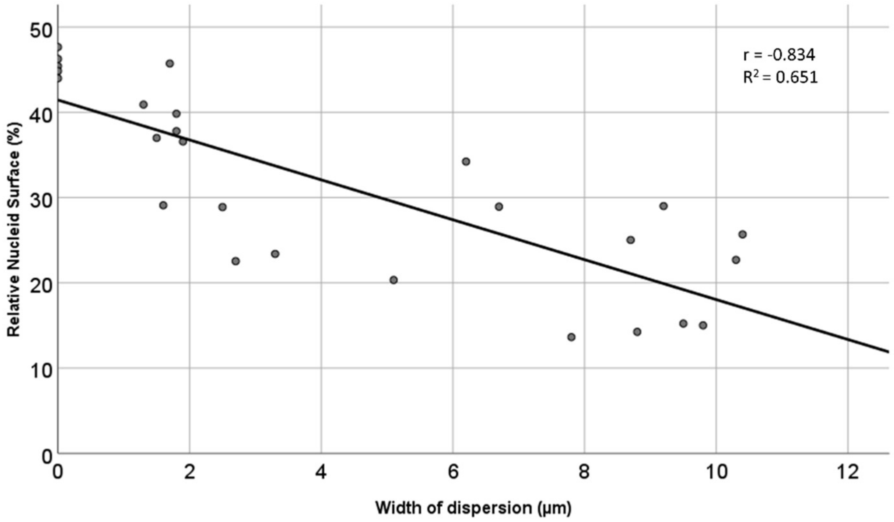

Figure S1: Linear regression showing the relationship between the mean relative nucleoid surface (RNS) of *E. coli* strains incubated with ciprofloxacin and the mean diameter of dispersion of DNA spots observed after bacterial lysis

Supplementary Table S1

**RNS (%) (Mean ± SD)**		**Ciprofloxacin (mg/L)**			
		0	0.06	0.25	0.5
**Ampicillin (mg/L)**	0	70.34 ± 3.71	38.82 ± 5.60	30.03 ± 5.29	23.19 ± 3.67
	1	71.34 ± 3.61	38.14 ± 5.11	31.00 ± 4.93	22.87 ± 3.56
	2	71.09 ± 3.52	38.71 ± 4.51	30.72 ± 4.99	23.61 ± 4.30
	4	71.01 ± 2.38	38.96 ± 5.28	31.01 ± 4.75	23.01 ± 3.29
	8	70.49 ± 2.97	39.78 ± 5.80	30.69 ± 5.07	22.96 ± 3.45

Supplementary Table S2.

DNA fragments/µm^2 (Mean ± SD)		Ampicillin (mg/L)			
		0	2	4	8
Ciprofloxacin (mg/L)	0	0.07 ± 0.04	0.53 ± 0.28	1.48 ± 0.29	2.10 ± 0.38
	0.06	0.08 ± 0.04	0.53 ± 0.23	1.38 ± 0.40	2.13 ± 0.50
	0.125	0.11 ± 0.08	0.47 ± 0.17	1.37 ± 0.44	2.06 ± 0.38
	0.25	0.09 ± 0.06	0.48 ± 0.16	1.41 ± 0.51	2.10 ± 0.43
	0.5	0.08 ± 0.05	0.42 ± 0.12	1.23 ± 0.25	1.63 ± 0.39
	1	0.09 ± 0.06	0.39 ± 0.11	0.96 ± 0.27	1.39 ± 0.33
